# Modification of SWCNTs with hybrid materials ZnO–Ag and ZnO–Au for enhancing bactericidal activity of phagocytic cells against *Escherichia coli* through NOX2 pathway

**DOI:** 10.1038/s41598-022-22193-1

**Published:** 2022-10-13

**Authors:** Osamah Al Rugaie, Majid S. Jabir, Mustafa K. A. Mohammed, Ruaa H. Abbas, Duha S. Ahmed, Ghassan M. Sulaiman, Salman A. A. Mohammed, Riaz A. Khan, Khalid A. Al-Regaiey, Mansour Alsharidah, Khalid M. Mohany, Hamdoon A. Mohammed

**Affiliations:** 1 Department of Basic Medical Sciences, College of Medicine and Medical Sciences, Qassim University, P.O. Box 991, Unaizah, 51911 Qassim Saudi Arabia; 2 Department of Applied Sciences, University of Technology, Baghdad, Iraq; 3 Department of Medical Physics, Al-Mustaqbal University College, 51001 Hillah, Babylon Iraq; 4 Collage of Dentistry, Al-Farahidi University, Baghdad, Iraq; 5 Department of Pharmacology and Toxicology, College of Pharmacy, Qassim University, Qassim, 51452 Saudi Arabia; 6 Department of Medicinal Chemistry and Pharmacognosy, College of Pharmacy, Qassim University, Qassim, 51452 Saudi Arabia; 7 Department of Physiology, College of Medicine, King Saud University, Riyadh, Saudi Arabia; 8 Department of Physiology, College of Medicine, Qassim University, Buraydah, 51452 Saudi Arabia; 9 Department of Medical Biochemistry and Molecular Biology, Faculty of Medicine, Assiut University, Assiut, 71515 Egypt; 10 Department of Pharmacognosy and Medicinal Plants, Faculty of Pharmacy, Al-Azhar University, Cairo, 11371 Egypt

**Keywords:** Biological techniques, Biotechnology, Drug discovery, Microbiology, Materials science, Nanoscience and technology

## Abstract

Zinc oxide-silver (ZnO–Ag), and zinc oxide-gold (ZnO–Au) nano-composites were prepared through wet chemical process and laced into single-walled carbon nanotubes (SWCNTs) to yield ZnO–Ag-SWCNTs, and ZnO–Au-SWCNTs hybrids. These nano-composite-laced SWCNTs hybrids were characterized using Raman spectroscopic, X-ray diffraction (XRD), scanning electron microscopy (SEM), and transmission electron microscopy (TEM) analyses. The hybrids were evaluated for their effects on phagocytic cells and bactericidal activity against the gram-negative bacteria *E. coli.* Their phagocytic cell activities and intracellular killing actions were found to be significantly increased, as the ZnO–Ag-SWCNTs and ZnO–Au-SWCNTs nano-hybrids induced widespread clearance of *Escherichia coli.* An increase in the production of reactive oxygen species (ROS) also led to upregulated phagocytosis, which was determined mechanistically to involve the phagocyte NADPH oxidase (NOX2) pathway. The findings emphasized the roles of ZnO–Ag- and ZnO–Au-decorated SWCNTs in the prevention of bacterial infection by inhibiting biofilm formation, showing the potential to be utilized as catheter coatings in the clinic.

## Introduction

Catheter-associated urinary tract infections (CAUTIs) are among the most common nosocomial infections that lead to patients becoming critically ill and extended hospital stays. Notably, prolonged catheterization plays a role in increased treatment expenses, morbidity and mortality. Almost 80% of healthcare-related urinary tract infections (UTIs) are CAUTIs^1^. Additionally, approximately 2–4% of these cases may lead to the development of bacteraemia, wherein *Escherichia coli* (*E. coli*) is the most common pathogen that causes CAUTI^2^. *E. coli* can form biofilms and propagate from there. The microorganisms isolated from patients with CAUTIs are more resistant to antibiotics than the microbial organisms isolated from patients with UTIs. Extended-spectrum beta-lactamase (ESBL)-producing *E. coli* have been isolated from catheter biofilms, which display more advanced antibiotic resistance than UTI-based *E. coli* isolates. In this context, it has been suggested that the antibiotic resistance developed by *E. coli* in the urinary catheter can be controlled by internally and externally coating the catheter tube with an antibiotic preparation. The use of zinc-, silver-, and gold-based nanomaterials has been reported to present antibacterial activities against a number of pathogens^3,4^. To couple the strongly resistant ESBL class of *E. coli* strains, superior antibiotic materials are needed, and a combination of zinc-, silver-, and gold-based nano-composites in hybrid preparations with single-walled carbon nanotubes (SWCNTs) as support-based nano-composites has been suggested.

Carbon nanotubes (CNTs) have several unique properties that have not been observed in other nanomaterials, including a large surface area, a high adsorption capacity, and thermochemical stability^5^, which makes them seemingly suitable for use as the base material for elemental nano-composite layering, coating, and adsorption. Despite their known cytotoxicity and low water solubility^6^, carbon nanotubes (CNTs) have been oxygenated (O2) to reduce toxicity, increase solubility, and provide interacting capabilities with structures and organisms^7^. The oxygen functionality attached to the CNT is important for developing nano-hybrid materials that may feasibly pass through the cell wall without causing any adverse biological responses and intracellularly transport chemo-biological agents, which may also include antibiotics^8^. Nonetheless, the interactions between CNTs and the immune system components are of significant concern^9^. Among the types of immune cells, macrophages are the phagocytic populations that open to meet CNTs after their planned administration or occupational exposure to individuals. Macrophages have recently been identified as target cells in the treatment of inflammation-associated complications, such as tumours and atherosclerosis^10^. For the reasons stated above, CNT-macrophage interactions have been extensively studied to better understand macrophage behaviour. Moreover, CNTs have been documented as the single most hazardous stimulus to macrophages, which results in inflammation and fibrosis^11,12^. Macrophages exhibit potent reactions during developing responses, as evidenced by their beneficial involvement in host defence regulation, inflammation, and homeostasis^13^. Owing to their powerful capacities to engulf bacterial entities, present antigens, and release cytokines, these cells can regulate the initiation and resolution phases of both the innate and adaptive immune responses^14^. The immune-regulatory, inflammatory, and proliferative capabilities, as well as cellular metabolism and tissue remodelling abilities, are among the most important functions of macrophages in various states. Macrophages also play an important role in recognizing, processing, and clearing xenobiotic and non-natural nanomaterials under in vivo conditions^15^ and work in tandem with micro-milieu stimuli that polarize macrophages into one of two phenotypes^16–19^. The interaction of nanoparticles (NPs) with these cells has sparked much interest in medical applications^20^. Hence, the variety of in vivo biological effects of nanomaterials and the development of nanomaterial-based therapeutics has necessitated a better understanding of the role of nanomaterials in macrophage functionalization^21,22^.

In recent years, it has been established that macrophages are malleable and may be triggered in a variety of ways, allowing them to perform a variety of functions depending on the stimulus they receive from their surroundings. As a result, these contradictory clues point to macrophage cells being stimulated by CNTs with a distinctive activation behaviour rather than a simple immunosuppressive action. To the best of our knowledge, there is minimal existing information regarding macrophage stimulation and its related functions when these cells are exposed to CNTs. Therefore, this study aimed to measure the activity of phagocytic cells against *E. coli* mediated by prepared hybrid nanomaterials. In this context, ZnO–Ag-SWCNTs and ZnO–Au-SWCNTs were prepared as nano-hybrid materials. Zinc oxide (ZnO) and metal (Ag and Au)-doped ZnO NPs have recently been observed to be among the most active antibacterial nanomaterials^23,24^ and have shown biocompatibility in a broad range of cells^25–27^. Furthermore, the biomedical applications of CNTs have piqued the interest of researchers. In this study, ZnO–Ag-SWCNTs and ZnO–Au-SWCNTs preparations were used to explore the effects of these nanomaterials on macrophage activity to find their plausible utility as antibacterial agents for certain biological purposes and clinical uses, including as a coating material feasible for catheter applications. The results showed that ZnO–Ag-SWCNTs and ZnO–Au-SWCNTs induced strong bactericidal activity against *E. coli* through a reactive oxygen species (ROS)-dependent mechanism in catheter materials.

## Results and discussion

### XRD diffraction analysis

**Figure 1:**
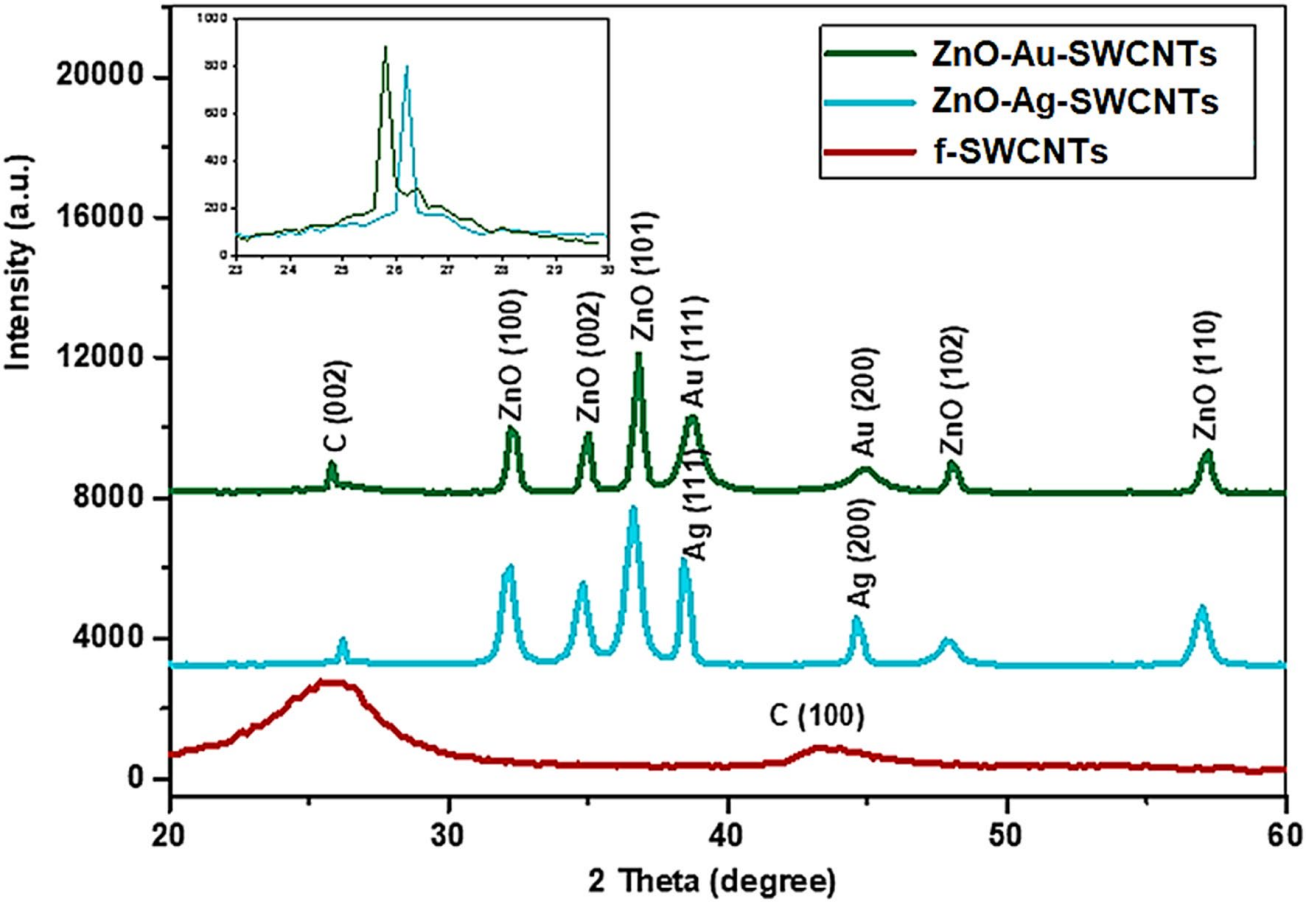
XRD pattern of the ZnO–Au-SWCNTs, ZnO–Ag-SWCNTs, and *f*-SWCNTs.

X-ray diffraction (XRD) analysis was utilized to identify the crystallinity and crystallite size of the prepared samples. Figure 1 shows the X-ray powder-intensity-functionalized (*f*-SWCNTs) and ZnO–Ag-SWCNTs and ZnO–Au-SWCNTs nano-hybrids. The X-ray spectra of the functionalized SWCNTs revealed a broad and dominant peak at 2θ = 25.4° and a low-intensity peak at 2θ = 44.8° for diffraction planes (002) and (100), respectively. These peaks are associated with the graphite sheets in the SWCNT structure and correspond well to previously published data^8^. The interlayer distance (d) for the (002) plane was determined to be ~ 0.35 nm by using Bragg’s law, which is consistent with the standard spacing between graphite layers (0.34 nm).

$$n\lambda =2d \sin\theta (\mathrm{Bragg's} \, \mathrm{Law})$$

Here, λ, θ, and n are the X-ray wavelength (0.1546 nm), diffraction angle, and diffraction peak order, respectively. The X-ray spectra of the ZnO–Ag-SWCNTs and ZnO–Au-SWCNTs nano-hybrids demonstrated the polycrystalline nature of the ZnO and metallic NPs (Ag and Au, i.e., ZnO–Ag and ZnO–Au) with hexagonal Wurtzite and face-centred cubic phases, respectively (Fig. 1). The peaks at 2θ = 31.3°, 34.4°, 36.3°, 47.8°, and 56.8° corresponded to the (100), (002), (101), (102), and (110) planes, respectively, for ZnO. Another two peaks at 2θ = 38.13°, and 44.3° were ascribed to the (111) and (200) planes of the silver and gold NPs. These observations were consistent with previously reported data^27^. The Debye–Scherrer law was used to calculate the average crystallite size (*D*) of the Ag NPs and NPs loaded onto the SWCNTs.

$$D=\frac{k \lambda }{B \cos\theta } (\mathrm{Debye}-\mathrm{Scherrer's} \, \mathrm{Law})$$

Here, *B* and *k* are the full widths at half maximum and Scherrer constant (0.9), respectively. The crystallite sizes of Au and Ag were deduced to be 13 to 15 nm, respectively. Moreover, the X-ray spectra of the nano-composites displayed peaks at 2θ = 26.1° for the ZnO–Ag-SWCNTs and 2θ = 25.9° for the ZnO–Au-SWCNTs, which were ascribed to the (002) diffraction plane of the SWCNTs in the prepared nano-hybrid materials. The corresponding d-spacing was equal to 0.341 nm and 0.343 nm for the ZnO–Ag-SWCNTs and ZnO–Au-SWCNTs, respectively. As observed, the (002) diffraction peak of the SWCNTs in the nano-composites was displaced to a higher angle compared with the functionalized SWCNTs (Fig. 1). These observations indicated that the d-spacing decreased after ZnO–Au and ZnO–Ag were loaded onto the SWCNTs. The high impacts of the ZnO NPs on the SWCNTs were thought to have caused these displacements.

### Raman spectroscopic analysis

**Figure 2:**
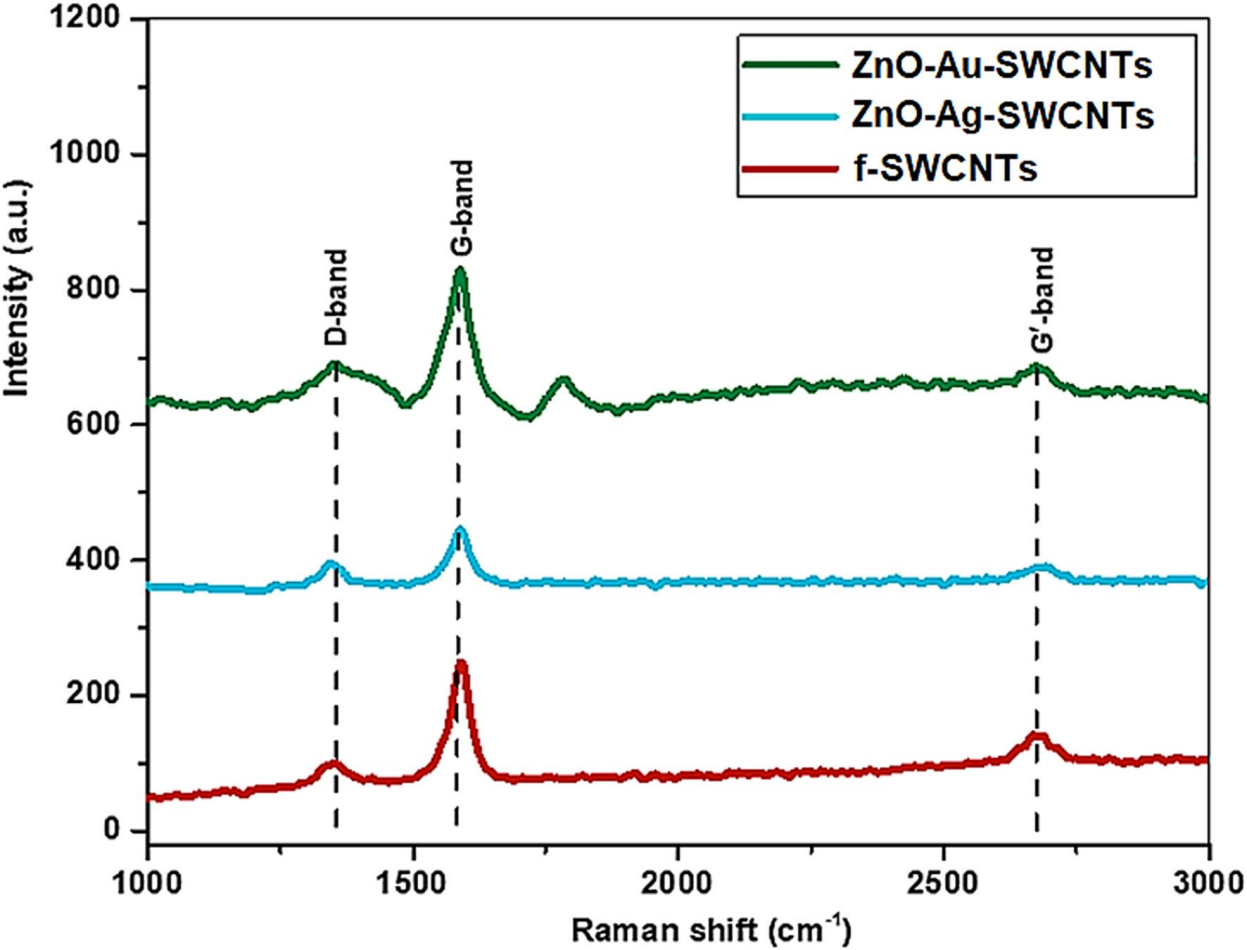
Raman spectra of ZnO–Au-SWCNTs, ZnO–Ag-SWCNTs, and *f*-SWCNTs.

Raman spectroscopy is a suitable tool for the investigation of CNTs, which have three unique bands. These bands are the disorder band (D), associated with *sp*^3^ defects, the tangential band (G) due to the in-plane stretching of graphitic rings with *sp*^2^, and the second-order band (G') originating from a double resonance of the disorder and defects. Figure 2 shows the Raman spectra for the *f*-SWCNTs, and the ZnO–Ag-SWCNTs and ZnO–Au-SWCNTs nano-hybrids. Band shifting to higher wavenumbers was observed for the nano-composites (ZnO–Ag and ZnO–Au) compared with the functionalized SWCNTs. Due to chemical functionalization and the presence of the composite NPs on the CNTs, sidewall defects were produced on the SWCNTs^28,29^. The intensity ratio of the D-band and the G-band (I_D_/I_G_) can be used to measure the level of disorder in the SWCNTs after the composite NPs are present. The increase in the I_D_/I_G_ ratio for the nano-composite materials compared with the functionalized SWCNTs is an indication of the anchoring of ZnO–Ag and ZnO–Au over the walls of the SWCNTs. The increased magnitude was attributed to the formed interactions of the energetic NPs, which led to damage to the CNT walls and an increasing number of defect sites.

### Morphological and chemical composition studies

**Figure 3:**
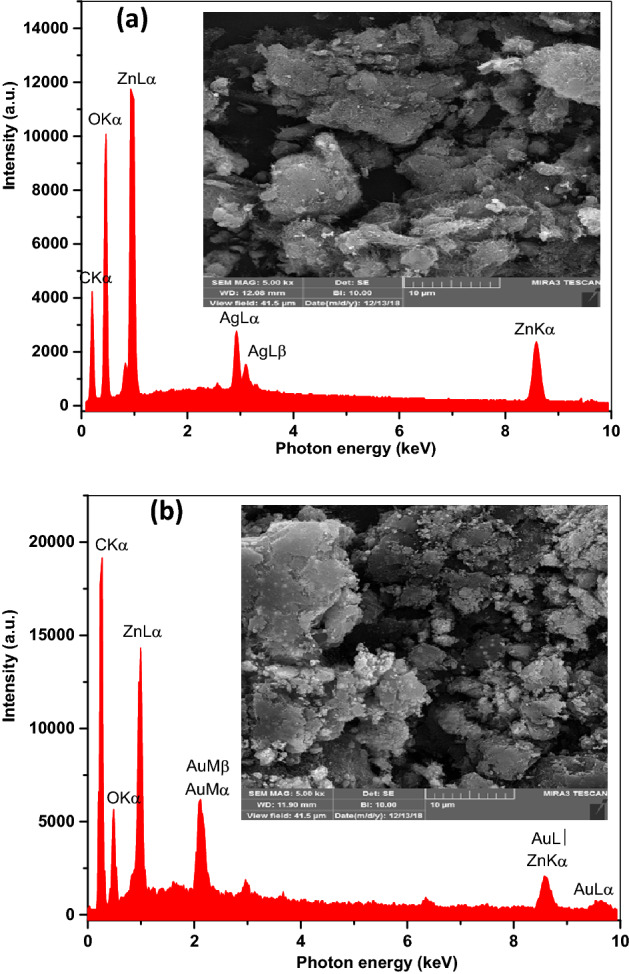
SEM and EDS analyses of, (**a**) ZnO–Ag-SWCNTs, and (**b**) ZnO–Au-SWCNTs.

**Figure 4:**
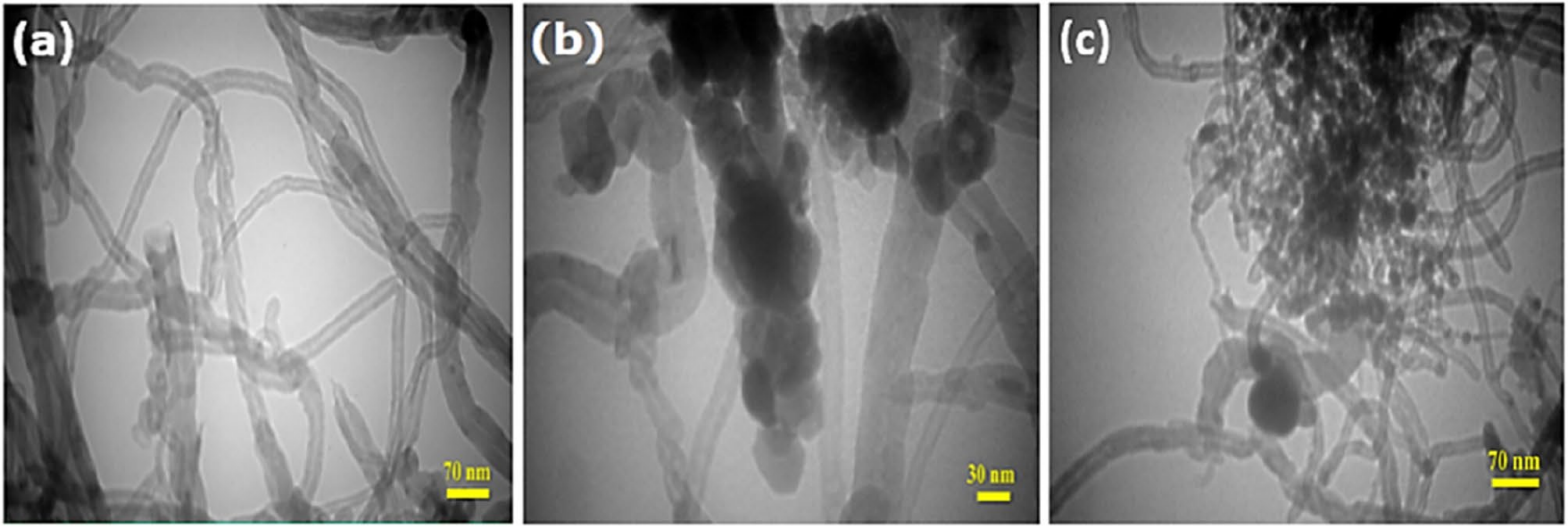
TEM images of (**a**) the *f*-SWCNTs, (**b**) ZnO–Ag-SWCNTs, and (**c**) ZnO–Au-SWCNTs.

Figure 3a and b exhibited the scanning electron microscopic (SEM) and energy-dispersive X-ray spectroscopy (EDS) analyses, which verified the presence of the ZnO-Ag and ZnO–Au NPs on the SWCNTs surface. The EDS spectrum of functionalized SWCNTs is shown in Fig. 3a^30^. Strong carbon and weak oxygen peaks were identified in the sample. It was therefore inferred from the EDS spectra of the ZnO–Ag-SWCNTs, and ZnO–Au-SWCNTs nano-composites that the oxygen groups were effectively attached to the surface of the SWCNTs. These spectra also suggested the presence of C, O, Zn, and Ag in the ZnO–Ag-SWCNTs and the presence of C, O, Zn, and Au in the ZnO–Au-SWCNTs nanomaterials. These findings revealed that the surfaces of the SWCNTs were coated with the ZnO–Ag and ZnO–Au nano-composite materials. Figure 3a and b also showed field-emission scanning electron microscopy (FESEM) micrographs (inset) of the ZnO–Ag-SWCNTs, and ZnO–Ag-SWCNTs at a magnification of 10 μm. Moreover, more detailed images and characteristics of the nano-structures of the prepared materials were visualized using transmission electron microscopy (TEM), as shown in Fig. 4a–c. The TEM images of the functionalized SWCNTs demonstrated fewer CNT aggregates with a diameter of 30 to 65 nm, as exhibited in Fig. 4a–c. Additionally, the TEM images of the ZnO–Ag-SWCNTs and ZnO–Au-SWCNTs nano-hybrids showed dark spherical shapes corresponding to the presence of the NPs, and the bright tubes corresponded to the SWCNTs. The sidewalls of the SWCNTs were coated with the NPs with a diameter of from 15 to 30 nm.

### The nano-hybrids increased bacterial intracellular killing

**Figure 5:**
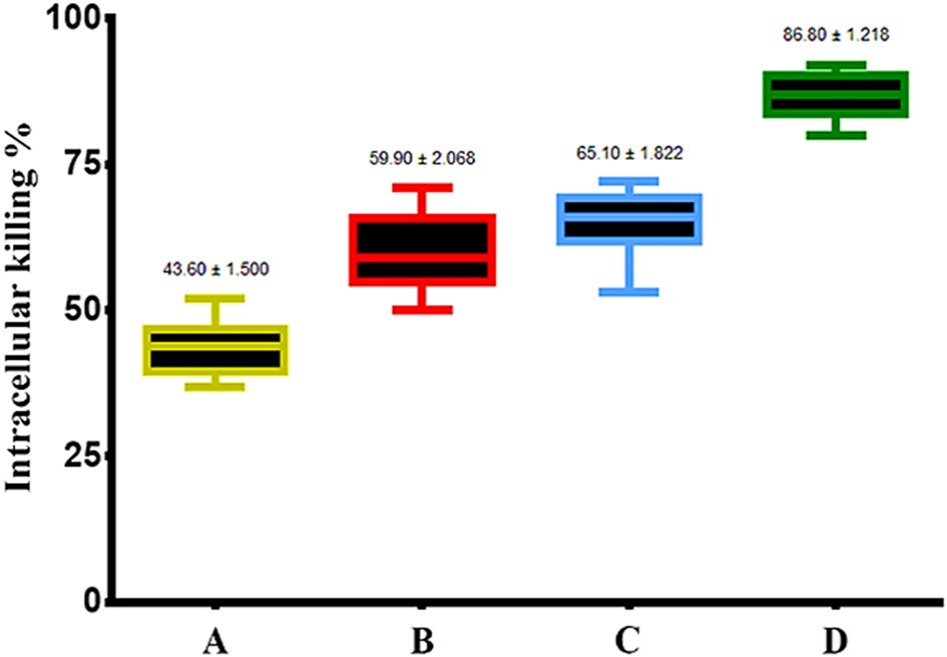
The prepared NPs increased the intracellular killing of *E. coli* by BMDMs. Data are presented as the mean ± SD. (**A**) Control BMDMs. (**B**) BMDMs treated with functionalized SWCNTs. (**C**) BMDMs treated with SWCNTs decorated with ZnO–Ag NPs. (**D**) BMDMs treated with SWCNTs decorated with ZnO–Au NPs.

The numbers of internalized *E. coli* in phagocytic cells (BMDMs) were investigated after infection in the presence or absence of SWCNTs and ZnO–Ag-SWCNTs and ZnO–Au-SWCNTs nano-hybrids to assess and compare the activities of the phagocytosis process and intracellular killing abilities. The ZnO–Ag-SWCNTs and ZnO–Au-SWCNTs nano-hybrid-pre-treated phagocytes showed noticeable increases in their ability to carry out intracellular killing of the ingested *E. coli* (Fig. 5). Thus, the SWCNTs and ZnO–Ag-SWCNTs and ZnO–Au-SWCNTs nano-hybrid-pre-treated phagocytic cells showed an increased bactericidal response to *E. coli*.

### Nano-hybrids induced phagosome maturation

**Figure 6:**
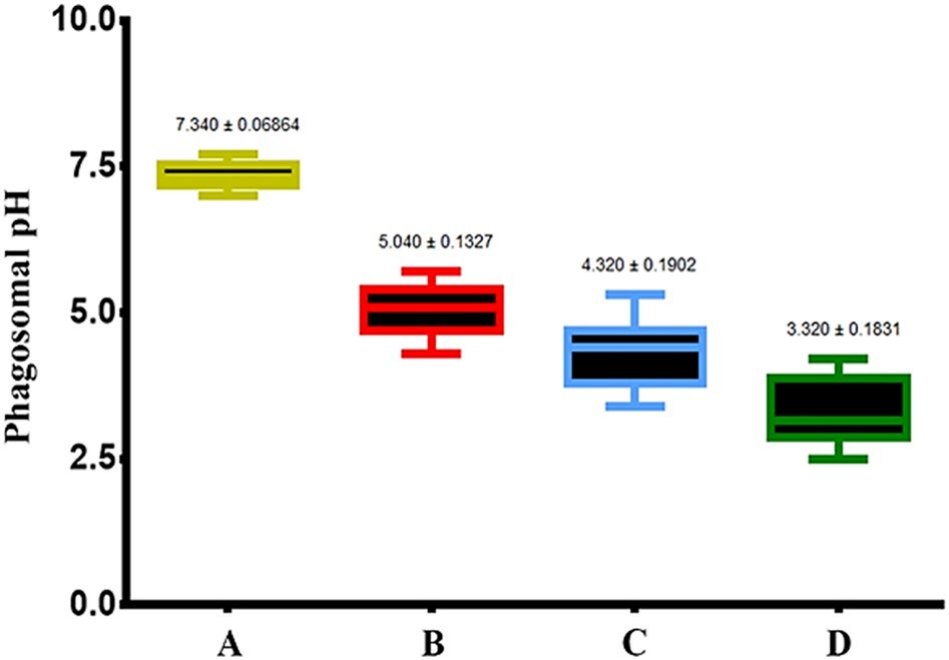
The prepared NPs increased phagosome maturation in BMDMs. Data are presented as the mean ± SD. (**A**) Control BMDMs. (**B**) BMDMs treated with functionalized SWCNTs. (**C**) BMDMs treated with SWCNTs decorated with ZnO-Ag NPs. (**D**) BMDMs treated with SWCNTs decorated with ZnO–Au-NPs.

**Figure 7:**
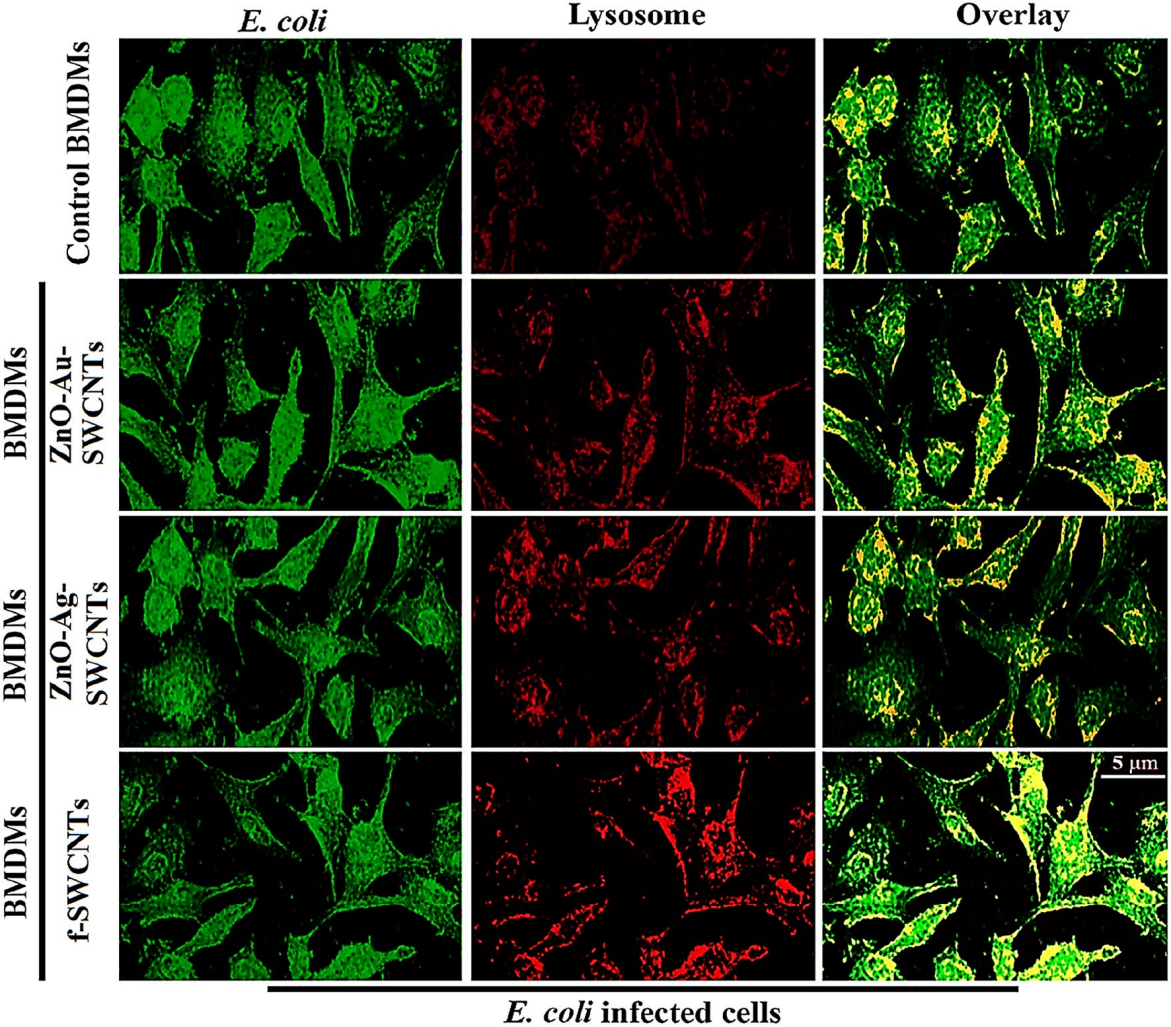
The prepared NPs increased the co-localization of the lysosomes and fluorescein isothiocyanate (FITC)-conjugated *E. coli*. co-localization appears as yellow, and lysosomes labelled with LysoTrackerRed® appeared red. Scale bar, 5 µm.

Phagocytes mature after the ingestion of bacteria, and this maturation is characterized by a decrease in phagosomal pH and phagosome-lysosome fusion activity^31^. The current work found a significant decrease in phagosomal pH after BMDM pre-treatment with the ZnO–Ag-SWCNTs and ZnO–Au-SWCNTs nano-hybrids and the ingestion of *E. coli* compared to control BMDMs (Fig. 6). The LysoTrackerRed®-loaded BMDMs selectively labelled the late endosomes/lysosomes and allowed examination of the maturation events of the *E. coli*-FITC-ingesting phagosomes by assessing their ability to co-localize with LysoTrackerRed®. This co-localization was demonstrated after 30 min in BMDMs pre-treated with SWCNTs and ZnO-Ag-SWCNTs and ZnO–Au-SWCNTs nano-hybrids. In parallel, most of the *E. coli*-FITC entities demonstrated smaller amounts of co-localization with LysoTrackerRed® in the control BMDMs (Fig. 7). These findings indicated that the BMDMs pre-treated with SWCNTs and ZnO-Ag-SWCNTs and ZnO–Au-SWCNTs nano-hybrids had enhanced phagosome maturation after the ingestion of *E. coli*.

### Nano-hybrids increased the phagocytosis of pHrodo *E. coli* bioparticles by BMDMs

**Figure 8:**
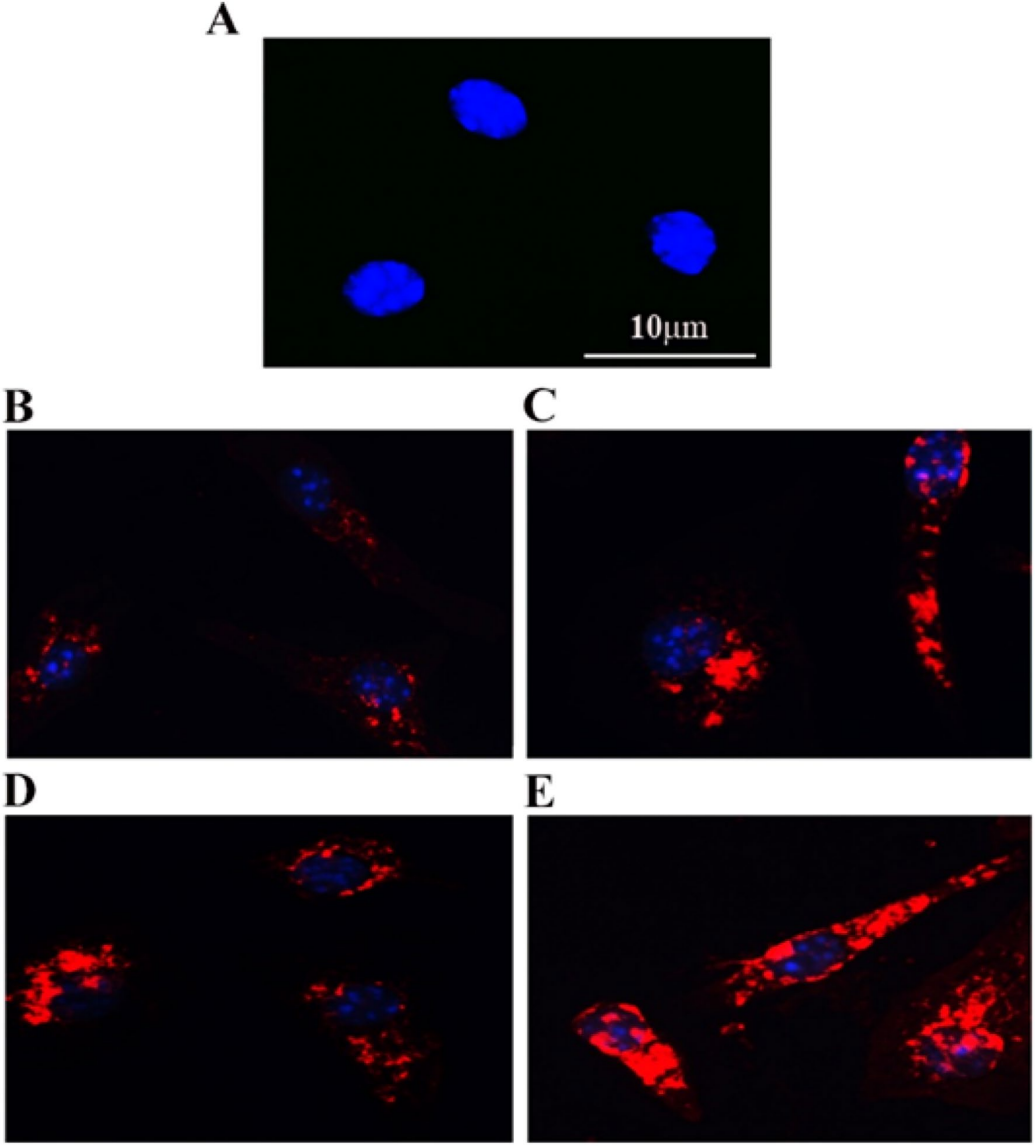
The prepared NPs improved the phagocytosis of pHrodo *E. coli* bioparticles. (**A**) Untreated control BMDMs stained with DAPI (blue). (**B**) BMDMs treated with pHrodo *E. coli* bioparticles (red). (**C**) Cells pre-treated with 10 µg/ml functionalized SWCNTs followed by treatment with pHrodo *E. coli* bioparticles. (**D**) Cells pre-treated with 10 µg/ml ZnO-Ag- followed by treatment with pHrodo *E. coli* bioparticles. (**E**) Cells were pre-treated with 10 µg/ml ZnO–Au-SWCNTs followed by treatment with pHrodo *E. coli* bioparticles.

The phagocytic activity of the BMDMs was measured by observing the uptake of tagged *E. coli*, which emitted fluorescence only after their transport into the lysosome (low pH) (Fig. 8). A comparison of the phagocytosis of the pHrodo *E. coli* bioparticles after BMDM pre-treatment with SWCNTs and the ZnO–Ag-SWCNTs and ZnO–Au-SWCNTs nano-hybrids was performed. The control BMDMs were found to have less phagocytosis potential than the BMDMs pre-treated with SWCNTs and the ZnO–Ag-SWCNTs and ZnO–Au-SWCNTs nano-hybrid materials.

### Nano-hybrids increased NOX2 in BMDMs

**Figure 9:**
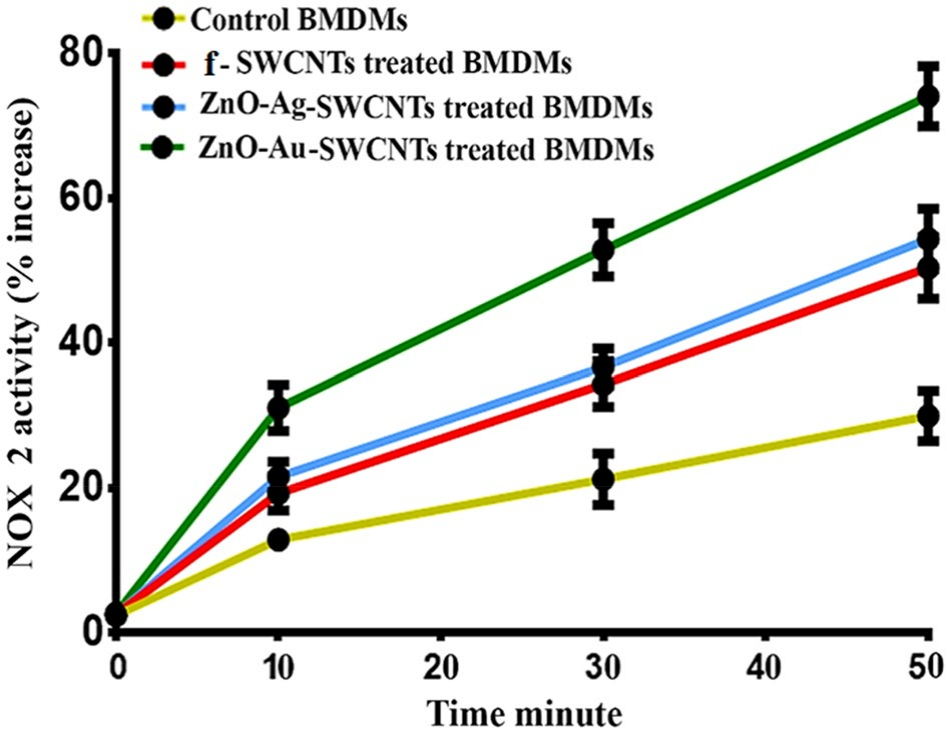
The prepared NPs increase NADPH oxidase 2 (NOX2) activity in BMDMs infected with *E. coli.*

Phagocytes have been reported to kill bacteria by activating the NADPH oxidase complex (NOX2) enzyme complex, which leads to superoxide anion (SOA) formation. These anions are generated by NOX2 in the membranes of phagosomes^32,33^. To evaluate the activities of NOX2 and ROS in BMDMs, SOA production by BMDMs was assessed. In comparison to the control BMDMs, the SWCNTs, ZnO–Ag-SWCNTs, and ZnO–Au-SWCNTs-pre-treated BMDMs exhibited noticeable increases in ROS and NOX2 activity with a significant release of SOA in response to the presence of *E. coli* (Fig. 9). Thus, the pre-treatment of the BMDMs with the ZnO–Ag-SWCNTs and ZnO–Au-SWCNTs nano-hybrids was inferred to cause the increase in NOX2 activity in response to enhanced ROS formation (a major mechanism of bacteria-killing clearance enhancement)^34^. Although other mechanisms, such as cell wall damage^35^ and DNA/RNA destruction^36^, cannot be ruled out, they were not tested in the current study. Furthermore, bacteria trapping in aggregated nanomaterials (both ZnO–Ag and ZnO–Au nano-composites, as well as ZnO–Ag-SWCNTs and ZnO–Au-SWCNTs nano-hybrids), as well as cell wall damage caused by the sharp edges of the NPs, cannot be ruled out^37,38^.

Previously, a wet chemical method was used to prepare SWCNTs decorated with ZnO–Ag and ZnO–Au as nano-composite materials. These prepared materials showed an increase in the activity of phagocytic cells against *Staphylococcus aureus.* The SWCNTs decorated with ZnO–Ag and ZnO–Au markedly increased the bacterial intracellular killing and induction of the clearance of *S. aureus,* and that was corroborated by a significant increase in NOX2 activity^39^.

In another study, the gold nanoparticles (GNPs) and graphene oxide flakes (GOFs) significantly aided the phagocytosis activity of phagocytic immune cells against Gram-positive and Gram-negative human pathogenic bacteria, *S. aureus* and *E. coli.* The combined GNPs and GOFs induced significant clearance of bacteria through phagosome maturation and production of reactive oxygen species through NOX2 pathway was reported^40^.

The raw-SWNTs and raw-MWNTs were chemically oxidized with a mixture of sulphuric acid (H_2_SO_4_), and nitric acid (HNO_3_) under ultrasonic vibrations. The antibacterial effects of the SWCNTs and MW-CNTs against *E. coli* were evaluated through the viable count method, and fluorescence microscopy. The results of the viable count method showed that the SWCNTs and MWCNTs have higher inhibitory effects after being treated with H_2_SO_4_/HNO_3_^41^.

### Anti-biofilm activity

**Figure 10:**
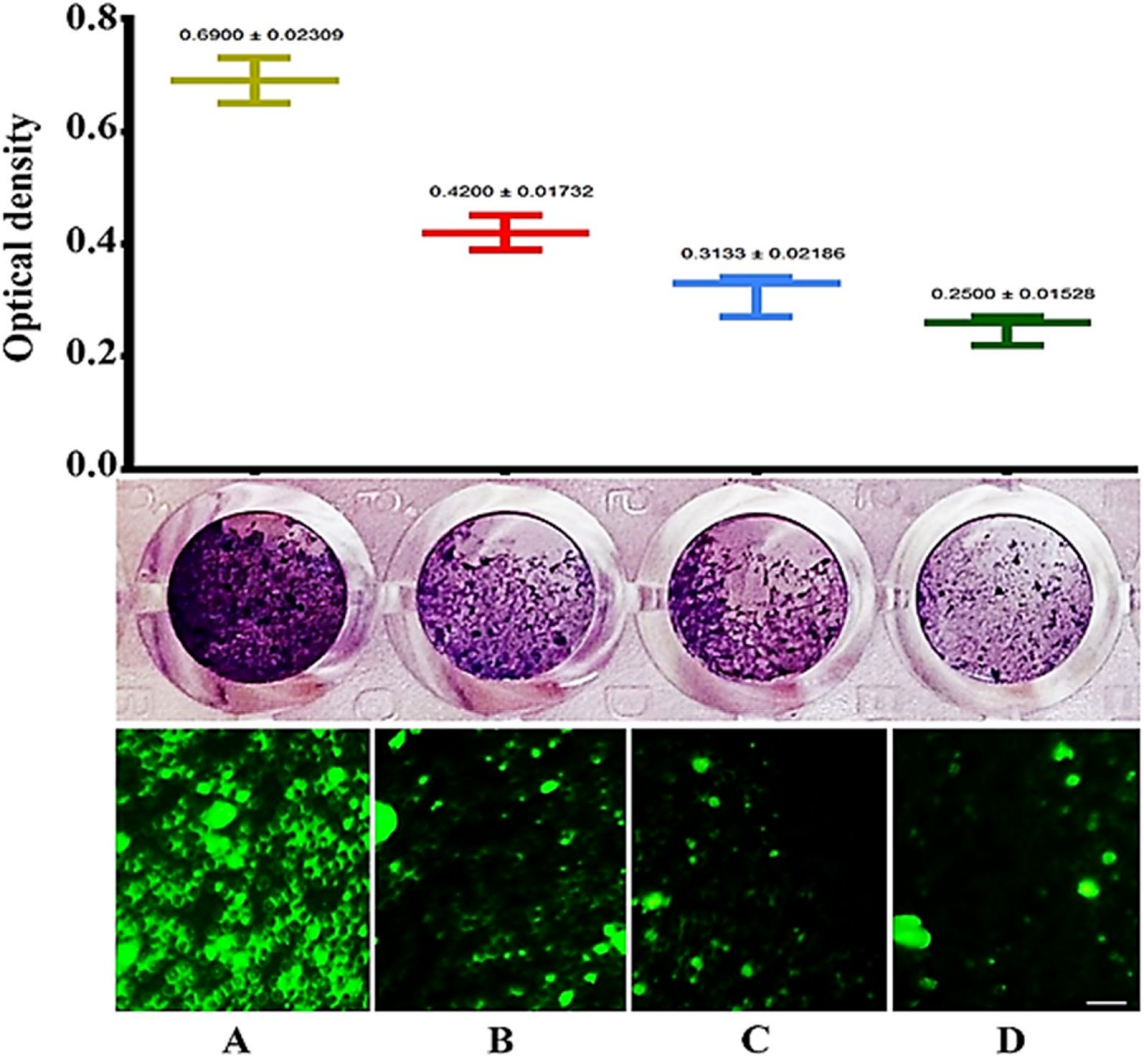
SWCNTs decorated with ZnO–Ag and ZnO–Au reduce *E. coli* biofilm formation. The upper panel represents the quantification of biofilm formation as determined by crystal violet staining. The lower panel shows fluorescence microscopy images. A nucleic acid stain was used. The scale bars represent 10 µm. (**A**) Untreated control *E. coli*. (**B**) *E. coli* treated with functionalized SWCNTs. (**C**) *E. coli* treated with ZnO–Ag-SWCNTs. (**D**) *E. coli* treated with ZnO–Au-SWCNTs.

The adhesion of *E. coli* on surfaces is an essential step in the initiation of any infection. *E. coli* has the ability to form biofilms after adhering to different types of surfaces, such as live cells and medical equipment, e.g., urinary catheters^42–44^. Staining adherent bacterial cells with crystal violet has been used to detect bacterial biofilms (30). All of the nano-hybrids reported here showed activity against biofilm formation (Fig. 10). In comparison to the untreated control *E. coli*, SWCNTs, ZnO–Ag-SWCNTs, and ZnO–Au-SWCNTs pre-treated *E. coli* showed significant reductions in biofilm formation (0.69, 0.42, 0.31 and 0.25 OD_595_, respectively). These reductions were probably caused by the formation of ROS, which ruptured the bacterial cells^45^. Fluorescence microscopy (Fig. 10) showed that the *E. coli* cells adhered to the surfaces of the untreated chamber slide and formed a mature biofilm within 24 h of incubation. The formation of these biofilms decreased in the wells treated with ZnO–Ag-SWCNTs and ZnO–Au-SWCNTs at a concentration of 250 µg/ml for 24 h. We, therefore, conclude that ZnO–Ag-SWCNTs and ZnO–Au-SWCNTs prevent biofilm development at a stage subsequent to the initial surface attachment and continue to inhibit biofilm growth^45,46^.

## Materials and methods

### Functionalization of the SWCNTs

First, 150 ml of sulphuric acid was used to chemically modify 0.5 g of raw SWCNTs (98%, diameter 5–30 nm, length 5–12 nm) to create oxygen-functionalized SWCNTs. The mixture was ultra-sonicated for 15 min at 30 °C, diluted with absolute ethanol, filtered through a polycarbonate membrane with a pore size of 0.22 µm, and dried for one day at 80 °C.

### Preparation of the ZnO–Ag-SWCNTs nano-hybrids

ZnO-Ag-SWCNTs nano-composites were prepared by a chemical precipitation technique. In 100 ml of acetone, 1 g of zinc acetate and silver nitrate (Ag:Zn, 5%, molar ratio) were dispersed. Under ultra-sonication for 10 min, the previously functionalized SWCNTs (0.1 g) dispersion was added, followed by the gentle addition of 0.5 g of oxalic acid (dispersed in 100 ml of acetone) to the mixture and stirring at 50 °C for 2 h. Finally, the mixture was dried for 24 h at 100 °C and annealed for 2 h at 400 °C.

### Preparation of the ZnO–Au-SWCNTs nano-hybrids

Briefly, 200 mg of ZnO NPs and 100 mg of functionalized SWCNTs were dispersed in 100 ml of distilled water (DW), and 40 mg of gold chloride (Au:Zn, 5%, molar ratio) in 100 ml of DW was added and the mixture was boiled. The ZnO-SWCNTs dispersion was added to the gold nanoparticle (Au NP) mixture and stirred for 2 h, followed by the addition of 20 ml of 0.01 M tri sodium citrate (aqueous) under stirring for an additional 1 h. The solution was then left to precipitate for 24 h. The formed precipitate was washed with DW and ethanol and filtered remove any impurities. Finally, the resulting sample was dried at 120 °C for 12 h to obtain the nano-hybrid powder.

### Bone marrow-derived macrophages (BMDMs)

Male C57/BL6 mice (7–8 weeks old) were used as the source for the isolation of primary BMDMs as previously reported^47^.

### Intracellular bacterial killing assays

The clinical isolate *E. coli* strain used in the present study was obtained from patients with UTIs admitted to Medical City in Baghdad, Iraq. The isolate was processed and identified by standard biochemical methods at the Microbiology Laboratory, Division of Biotechnology, Department of Applied Sciences, University of Technology, Baghdad, Iraq. The isolated *E. coli* were cultured in LB broth and incubated at 37 °C until the mid-log phase. The optical density (OD) was measured with a spectrophotometer at 550 nm and the absorbance value was ~ 0.4–0.6. After centrifugation at 3500 rpm for 15 min, the samples were washed 3 times using phosphate-buffered saline (PBS; pH 7.2). The OD of the suspended *E. coli* was measured with a spectrophotometer (OD_550_, ~ 10^8^ CFU/ml). The BMDMs were used alone or were pre-treated with ZnO-Ag-SWCNTs, or ZnO–Au-SWCNTs at a concentration of 10 µg/ml. The BMDMs were then infected with *E. coli* at an MOI of 1:100 (incubated for 90 min at 37 °C). Lysis buffer was then added to the sample to lyse the BMDMs. Serial dilutions of the lysates were cultured on LB agar plates overnight to assess the total and extracellular bacterial killing values, which were used to derive the intracellular bacterial killing values.

### Determination of the phagosomal pH

The luminal pH of the phagosome was determined^40^. In brief, double labelling of *E. coli* (heat-killed) was performed with 5 mg/ml carboxy-fluorescein-succinimidyl ester, a fluorescent probe with pH sensitivity, and 10 mg/ml carboxy-tetraethyl rhodamine-succinimidyl ester, a fluorescent probe with no sensitivity to pH (Molecular Probes Inc., Eugene, OR, USA). Afterwards, a pulse process for the isolated BMDMs was performed with the labelled bacteria, MOI = 1:50, for 30 min of incubation at 37 °C in the presence or absence of the ZnO–Ag-SWCNTs and ZnO–Au-SWCNTs hybrid materials. The phagosomal pH was determined using a standard curve based on the fluorescein/rhodamine fluorescence ratio.

### Phagosome/lysosome fusion assays

BMDMs were isolated and plated in 4-well chamber slides at a concentration of 1 × 10^5^ cells/ml in RPMI-1640. Ten hours later, the BMDMs were treated with the ZnO-Ag-SWCNTs and ZnO–Au-SWCNTs nano-hybrid materials (10 µg/ml) for 1 h. The cells were loaded with LysoTrackerRed® (25 nM) at 37 °C for 60 min before incubation with fluorescein isothiocyanate (FITC)-conjugated *E. coli* (MOI = 1:50) for 2 h. During the infection period, LysoTrackerRed® was introduced. The cells were washed five times with cold sterile PBS before being fixed with 4% paraformaldehyde (PFA). A fluorescence microscope was used to examine the samples (Olympus, Tokyo, Japan). Unfused phagosomes harbouring FITC-labelled bacteria showed green staining and LysoTrackerRed®-labelled lysosomes showed red staining. Due to the fusion of the 2 tagged fluorochromes, the phagosomes and lysosomes appeared yellow.

### Phagocytosis of pHrodo *E. coli* bioparticles by BMDMs

BMDMs were isolated and plated in a 4-well plate. The cells were pre-treated with the ZnO–Ag-SWCNTs and ZnO–Au-SWCNTs nano-hybrids (10 µg/ml). Next, 1 × PBS (pH 7.4) was added to the pHrodo-particles followed by vortexing, and 50 µl of the buffer solution was transferred to the cells. After 2 h, the cells were fixed and stained with DAPI, and photos were taken at 400 × magnification using a Zeiss confocal microscope. The blue dots represent the nucleus of the cells, whereas the red dots represent the *E. coli*-pHrodo component in the phagocytes.

### NADPH oxidase assay

BMDM NADPH oxidase activity was determined by lucigenin (Bis-*N*-methyl acridinium nitrate) (Sigma–Aldrich, Milwaukee, WI, USA). BMDMs were incubated at 37 °C for various periods of time with heat-killed and opsonized isolates of *E. coli* (each separately with phagocytes: bacterial ratio, 1:100) in the presence and absence of 10 µg/ml ZnO–Ag-SWCNTs or ZnO–Au-SWCNTs.

### Crystal violet staining

*E. coli* (1 × 10^6^) were grown in 24-well plates and treated with ZnO–Ag-SWCNTs and ZnO–Au-SWCNTs at a concentration of 250 µg/ml for 24 h. The samples were then washed with PBS. Adhered *E. coli* were stained with 0.1% crystal violet followed by rinsing twice with DW. To quantify biofilm growth, 0.2 ml of ethanol (95%) was added to the crystal violet-stained wells for 2 h of incubation with shaking. The optical density was measured at 595 nm^48^.

### Fluorescence microscopy

*E. coli* were grown in a 4-well chamber slide and treated with ZnO–Ag-SWCNTs and ZnO–Au-SWCNTs at a concentration of 250 µg/ml for 24 h. The samples were washed and fixed using 2% PFA for 20 min at 37 °C. The samples were then permeabilized using 0.1% Triton X-100 for 20 min at room temperature (RT) and stained with SYTOX Green nucleic acid stain (SYTO® 9, Invitrogen-Molecular Probes Oregon) for 30 min. Finally, the cells were examined using a fluorescence microscope (Nikon, Tokyo, Japan).

### Statistical analysis

The experiments were carried out in triplicate. The data were processed and are presented as the mean ± SD using GraphPad Prism 8.0.2, San Diego, FL, USA. The P value was significant when ≤ 0.05^49^.

## Conclusions

The current study found that the presence of functionalized SWCNTs and pre-treatment of phagocytic cells with ZnO–Ag-SWCNTs and ZnO–Au-SWCNTs nano-hybrid materials enhanced phagocytosis by phagocytic cells. Mechanistically, the enhanced ROS and NOX2 production indicated at least one aspect of the bioaction procedure. Moreover, it was also established that the ZnO–Ag-SWCNTs and ZnO–Au-SWCNTs nano-hybrids contributed to the bactericidal activity against *E. coli* to a greater extent than the SWCNTs alone, as shown by the enhanced, excessive production of ROS, which is considered to be derived from increased NOX2 activation. It can therefore be concluded that SWCNTs and ZnO–Ag-SWCNTs and ZnO–Au-SWCNTs nano-hybrids are crucial for the proper functioning of the antibacterial response initiated by the host's innate immune system. It is also plausible that nano-hybrid materials that have strong antibacterial properties can be used as an antibacterial coating on catheters and can be a potential material for other clinical uses and applications in the biomedical field.

## Data Availability

All data are included in this published article.
